# RFID Label Tag Design for Metallic Surface Environments

**DOI:** 10.3390/s110100938

**Published:** 2011-01-17

**Authors:** Chong Ryol Park, Ki Hwan Eom

**Affiliations:** Department of Electronic Engineering, Dongguk University, 3-26, Pil-dong, Joong-gu, Seoul, Korea; E-Mail: parkcr@dongguk.edu

**Keywords:** RFID tag, metallic surface, supply chain, electric transformers

## Abstract

This paper describes a metal mount RFID tag that works reliably on metallic surfaces. The method proposes the use of commercial label type RFID tags with 2.5 mm thick Styrofoam103.7 with a relative permittivity of 1.03 attached on the back of the tag. In order to verify the performance of the proposed method, we performed experiments on an electric transformer supply chain system. The experimental results showed that the proposed tags can communicate with readers from a distance of 2 m. The recognition rates are comparable to those of commercial metallic mountable tags.

## 1. Introduction

Nowadays, the supply chain system is based on the use of bar codes for identification. However, for some time now there has been increased interest in the benefits of Radio Frequency Identification (RFID) systems. The efforts to reduce the cost, to expand the areas of application, to increase security, and to standardize have led to an increased market share for this technology [1].

RFID is a technology used for object identification, which finds various applications in retail, transportation, manufacturing and supply chains. RFID comprises readers, and transponders, also known as tags. Most RFID tags contain at least two parts. The first part is an integrated circuit for storing and processing information, modulating and demodulating a RF signal, and other specialized functions. The second part is an antenna for receiving and transmitting the signal. RFID tags are generally cheaper and simpler compared to active ones. They contain no batteries and can be fully encapsulated for ruggedness and protection.

The numerous potential applications of the RFID system make ubiquitous identification possible at frequency bands of 125 KHz (LF), 13.56 MHz (HF), and 860–960 MHz (UHF). Over the years, three key factors have driven a significant increase in RFID usage: decreased cost of equipment, better performance for reliable identification, and a stable international standard around the UHF band. As the usage of RFID systems has increased, manufacturers are pushing toward to higher operating frequency (UHF band) which has as a long reading range, high reading speed, multiple accesses capability, anti-collision, and small antenna size compared to LF or HF band RFID systems.

In supply chain management and factory automation, for example, it is possible to track the loaded vehicles (or conveyers) with goods by attaching a tag on them. Information on the vehicle and goods, such as the vehicle number, the contents of goods and time-stamped departure and location data, can be stored on the tag, and then read it at a specific time. The RFID tag and the RFID antenna are very sensitive to factors such as the reader type, tag position, and direction of the tag, material of the object, angle of the antenna, and speed of the object [1–4].

Passive UHF RFID tags are able to provide good reading ranges for object identification compared to LF or HF RFID tags, and they have relatively low cost, but the traditional passive RFID tags have under-performed in metal rich environments, limiting their utility. If an RFID tag is attached directly to metallic object, it may work poorly. The antenna performance is seriously decreased because of the reactance variation on the antenna impedance. Metal reflects the radio frequency of RFID tags, and therefore the tags either need to be specially designed metal or attached without touching the metal with special spacers. Many applications for supply chain and materials management require RFID tags with long read ranges, durability and reliable read-rates while mounted on a radio-interfering metal surface [5–10].

A number of commercial planar and label-like passive UHF RFID tags have been tested on a large aluminum plate. Test results have shown that as the tags are brought closer to the aluminum plate, the read range decreases. Various methods have been proposed by different researchers to solve the drawback of low recognition on metallic surfaces. A patch antenna with an electromagnetic band gap ground plane has been used in the tag design. Yu, Kim, and Son have offered different tag designs that use patch antennas [11]. In 2008 more than a dozen new passive UHF RFID tags emerged to be specifically mounted on metal. ODIN Technologies (Ashburn, VA, USA) produced a benchmark which showed varying performance of metal mount tags, with the greatest read distance being just over 25 feet in real-world conditions [12]. However, these tags are much more expensive and difficult to commercialize as they require new antenna and infrastructure [13].

In this paper, we propose a novel, economic and efficient method, which does not need new antennas, to use the lowest cost label tag, and capable of performing on a metallic surface. We executed two-experiments. First of all, the straight-line reading range capability was measured for the various RFID devices. Second, the reading ratio was checked by passing though a real input-output gate. In the second experiment a model of a transformer materials management method that is managed with manual processes or bar codes in the real field, and which gives inaccurate total remaining materials and also makes it hard to check the current materials status in real time was used. With the RFID system method proposed in this paper, the solutions of these problems will be verified with experiments. In these experiment, we used an ALR-9800-RFID device which is the major device on the RFID market. The RFID tag and its characteristics near metallic surface are detailed in Section 2. Section 3 presents a proposed RFID tag design, which also includes the simulation results. Section 4 shows an implementation and experimental results. Finally, some conclusions are presented in Section 5.

## 2. RFID Systems

**Figure 1.**
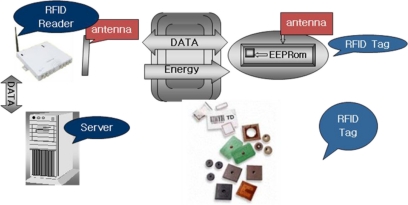
RFID System Structure.

In this section, we will discuss the inner workings of RFID systems. For the most part, an RFID system comprises three principal components, as shown in Figure 1.

The first is the tag, which is affixed to the item that is to be tracked or identified within the supply chain by the RFID system. The reader, which has a number of varied responsibilities including powering the tag, identifying it, reading data from it, writing to it and communicating with a data collection application. The data collection application receives data from the reader, enters the data into a database, and provides access to the data in a number of forms that are useful to the sponsoring organization.

An RFID system communicates with electromagnetic waves. When designing RFID tag antennas mountable on metallic platforms, it is very important to understand the behavior of the electromagnetic fields near metallic surfaces since the antenna parameters (the input impedance, gain, radiation pattern, and radiation efficiency) can be seriously affected by metallic platforms. In this section, the behavior of electromagnetic fields near metallic surfaces will be considered [5].

**Figure 2.**
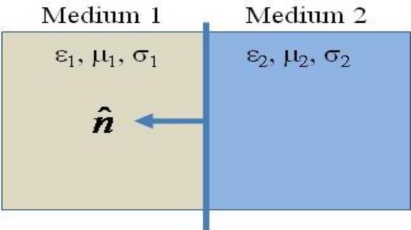
Boundary between two media.

We consider a boundary that lies between two media in space with medium 1 characterized by dielectric permittivity *ε*_1_, magnetic permeability *μ*_1_, and electric conductivity *σ*_1_, and medium 2 characterized by *ε*_2_, *μ*_2_, and *σ*_2_ (Figure 2).

If medium 1 is a metallic medium and we assume as a practical approximation that it is a perfect electric conductor with infinite conductivity, there will be no electric field in this medium. Consequently, D_1_ = 0, B_1_ = 0, and H_1_ = 0. Hence, for this case, the boundary condition become:

(5) $$\hat{n}\times E_{1}=0$$

(6) $$\hat{n}\cdot D_{1}=\rho_{s}$$

(7) $$\hat{n}\times H_{1}=J_{s}$$

(8) $$\hat{n}\cdot \;B_{1}=0$$

It is noticed that there are no tangential components of the electric field on a perfect electric conductor. On the other hand, there are only tangential components of the magnetic field directly next to a perfect electric conductor. Hence, not all components of electromagnetic fields are available near a perfect electric conductor.

## 3. Metal Mountable RFID Tag Design

**Figure 3.**
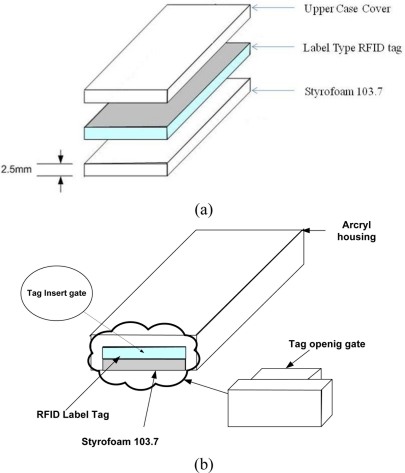
Proposed RFID tag design. **(a)** Side structure, **(b)** Front structure.

According to the theory of electromagnetic boundary conditions, there are only tangential components and no normal components of the magnetic field to the metallic surface. In addition, the magnetic field will be doubled when it is very near the metallic surface. The RFID tag design proposed here exploits this fact by having a gap between the metallic surfaces. The structure of the tag is shown in Figure 3.

**Figure 4.**
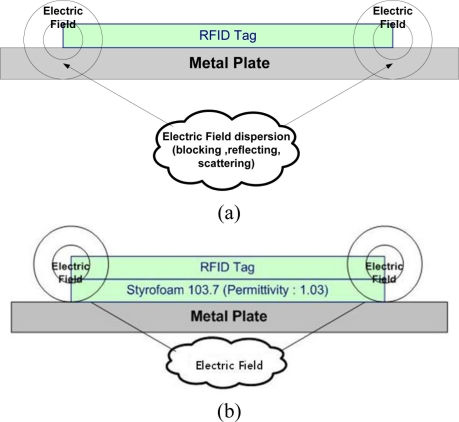
Electromagnetic field of metal plate and proposed tag. **(a)** Electromagnetic field of metal plate. **(b)** Electromagnetic field of proposed tag.

The proposed method is to use commercial RFID tags of label type, and Styrofoam103.7 material is attached on back side of the RFID tag. The Stryrofoam103.7’s thickness is 2.5 mm and its relative permittivity is 1.03. Using a dielectric material like Styrofoam makes the electromagnetic wave radiate on the top side of RFID tag. Figure 4 shows the electromagnetic field of the metal plate and proposed tag.

**Figure 5.**
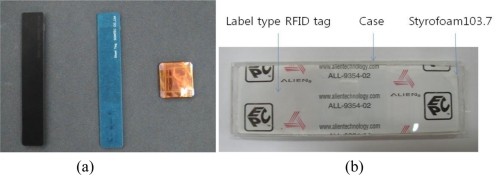
The photograph of commercial metal tags and proposed RFID design tag. **(a)** Commercial metal tags. **(b)** Proposed RFID design tag.

We compared proposed RFID tag with Sontec, Prenix, Smart1, and AWID tags which are commercial metallic mountable tags, and cost about $5 ∼ $10. We used a general purpose RFID tag, the ALL-9354-02 which costs about 5 cents, for the proposed RFID tag design. Figure 5 shows the photographs of the commercial metal tags and the proposed RFID design tag.

We tested the detecting range with ALR-9800 RFID-Reader, because we needed to estimate the optimum thickness of Styrofoam 103.7 before the comparison of a proposed structure with the commercial units.

**Figure 6.**
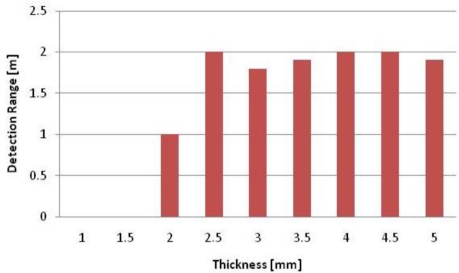
Results of Detecting range *vs.* Styrofoam thickness.

We measured the detection range between an RFID reader and the tag with varying Styrofoam103.7 thicknesses from 1 mm to 5 mm, as shown in Figure 6. The results showed that the RFID tag could be detected at 2 m range with a 2.5 mm thickness of Styrofoam 103.7.

## 4. Implementation and Experiments

**Figure 7.**
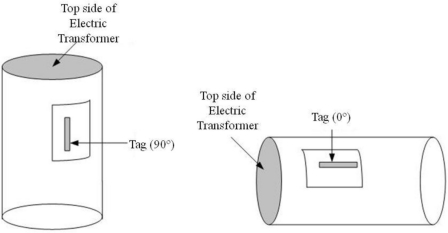
Attachment direction of RFID tags.

In this section, we show the test results comparing the performance of the commercial metal tags with the proposed RFID tag in a straight-line, and we verified the possibility of commercialization in an electric transformers supply chain management system. We attached metal tags and the proposed one on electric transformers with 90 (vertical) and 0 (horizontal) degree angles as seen in Figure 7.

**Figure 8.**
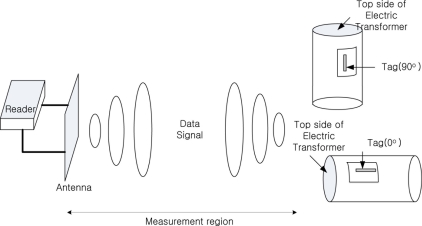
Installation for straight-line measurement experiments.

We measured the reading performance using the various tags and both fixed and hand-held readers as shown in Figure 8.

Experimental conditions were as follows:

- Tag : Metal Tag (Sontec, Prenix, Smart1, AWID), Label Tag (ALR’M’ Foam)
- Fixed type reader : ALR9800, Mecury4, LS, SAMSYS
- Handheld type reader : AWID, Hanmeg
- Antenna : ALIEN (Linear, Circular type Antenna), Others are Linear type Patch Antenna

Table 1 lists the detection range results over a straight-line. We can see that the proposed RFID label type tags can match the commercial unit reading range in this experiment.

**Figure 9.**
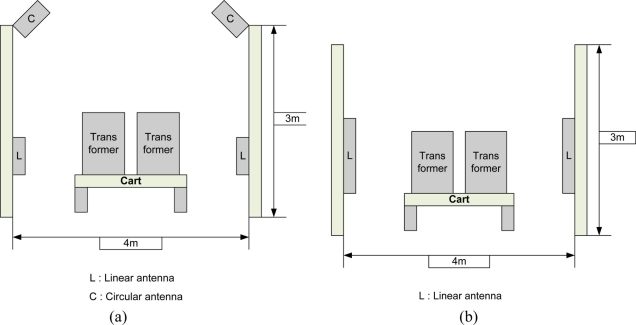
Experimental site set-up. **(a)** ALR-9800 RFID reader experiment. **(b)** Mecury4 RFID reader experiment.

All five kinds of experimental equipments showed at least a 2 meter commercial read range with both the proposed label type RFID tag and the commercial metallic tags. In addition, the recognition performance was good at zero degrees which the direction of tag was placed. In the tests, the ALR-9800 and Mecury 4 were the better of the devices. From the experimental result we could be sure that the proposed label type RFID tag showed good performance in addition to its low-cost. The second experiment was executed in a straight-line with real electric transformer. The set-up can be seen in Figure 9. We installed an asset management system which used the tags and a metallic material such as electric transformer, *etc.* The electric transformers have a metallic surface, and they pass through the warehouse gate as shown in Figure 9.

Experiment System Setup:

- Temperature: 0 ∼ 10 degree
- Humidity: 40 ∼ 60%
- RFID reader: ALR-9800, Mecury 4
- Antenna: ALR Patch Antenna (Circular, Linear), Mecury 4 Patch Antenna (Linear)
- The number of attached tags: 20 EA
- Iteration counts of experiment: 100
- Carton Speed: 0.5 m/s

**Figure 10.**
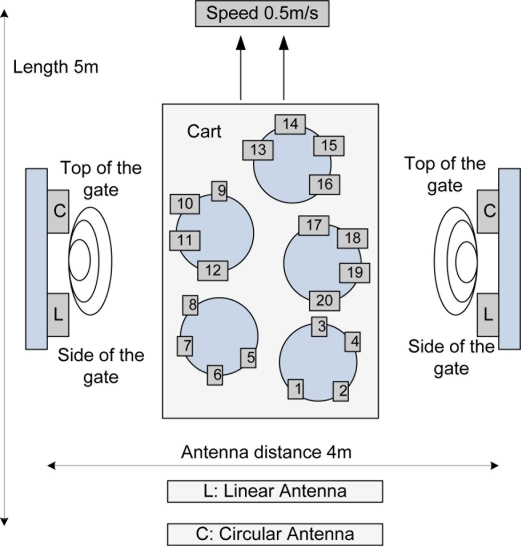
Multi-tag sensing experiment set-up.

**Figure 11.**
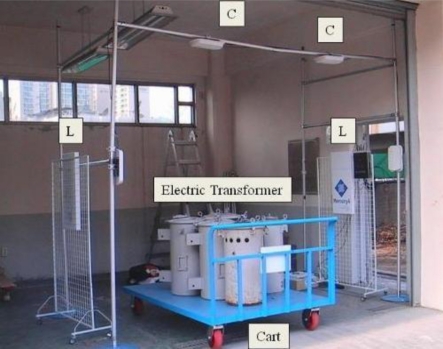
Photograph of the experimental site.

In the straight-line measurement test in which the space between ALR-9800 and the antenna of the reader was as given in Table 1, the performance of the linear antenna was good. According to this, the asset management gate should use the ALR-9800 or Mecury4 for improving the recognition ratio. The tags’ direction was vertical (90 degree). The multi-tag sensing experiment set-up is like Figure 10 and a photograph of the real-experiment is shown in Figure 11.

A total of 100 test iterations were executed with 20 tags using the set-up seen in Figure 11. Table 2 gives the recognition rate results for a Sontec metal tag and the proposed ALR ‘M’ Tag on foam label tag. Both tags’ reading ratio for a total of 100 test iterations was over 80%.

The results showed the difficulty of recognition to detect in metallic cylinder such as electric transformer, and the possibility of adoption to a real-world scenario. With these results we verified the performance of commercial metal tags and the proposed tag were similar in efficiency, but at a lower cost for the proposed tag.

**Figure 12.**
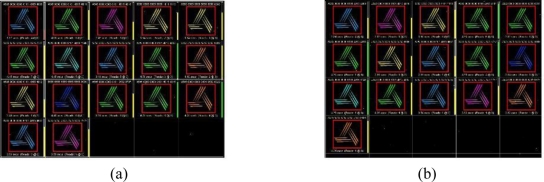
Screenshot of reading software. **(a)** Commercial metal tags. **(b)** Proposed RFID tags of label type.

Figure 12 shows the experimental results using both the proposed label type RFID tags and commercial metal tags obtained using ALR-9800 management software. From these results we were able to verify the similar performance between the proposed label type RFID tag and the commercial metal tag. It has been proved through the data provide in Tables 1 and 2 that the proposed RFID tag is similar to the commercial metal tags in performance but it is more economical, occupies less volume.

## 5. Conclusions

In this paper, we have described a metal mount RFID tag that works reliably on metallic surfaces. The proposed tag consisted of a commercial tag, and a material between tag and metallic surface. Styrofoam103.7 material, which has 2.5 mm thickness and 1.03 relative permittivity, was attached on back side of the RFID tag. The experimental results on an electric transformers supply chain show that the proposed tags can communicate with readers from a distance of 2 m. The recognition rates results are comparable to those achieved with more expensive commercial metallic mountable tags.

We verified that the efficiency performance of the commercial metal tags and the proposed tags were similar. The proposed tag is simple and economically advantageous. Furthermore, the proposed RFID tag does not show volume, price, and shape performance deviations, so it can be applied in the real world. In this paper, the proposed RFID tag showed the possibility of operating in the metal object field where application of RFID systems is difficult. We need to improve tag of efficiency through the reduction of recognition ratio as the moving the tag location, which affects the material handling. If the proposed RFID tag structure could achieve better durability and the stability, the RFID system could be used on different metal materials and applied in real use later.

## Figures and Tables

**Table 1.** Results of detection range (m).

**Tag**	**Reader angle**	**ALR9800**		**Mecury4**	**LS**	**AWID**	**Hanmeg ENG**	**SAMSYS**
		**Circular Antenna**	**Linear Antenna**					
**Sontec**	90	2.1	2.5	4.9	3.6	2	1.6	2.8
	0	2.3	2.6	6.3	3.7	2.8	2.1	3.8
**Prenix**	90	3.1	3.4	7.4	4.9	1.3	1.7	4.8
	0	3.3	3.7	8∼	5.1	1.8	3.4	5.4
**Smart1**	90	2.7	3.3	7.5	4.8	1.1	2.4	5.1
	0	3.0	3.4	8∼	5.0	1.0	3.9	6
**AWID**	90	2.8	3.2	5.6	4.1	1.7	3.8	5.1
	0	3.1	3.4	6.9	4.3	2.7	4.1	6.2
**ALR'M' Foam**	90	2.0	2.2	3.8	2.0	1.3	2.0	2.1
	0	2.2	2.3	4.8	2.1	1.0	2.2	2.2

**Table 2.** The recognition ratio results.

**RFID Tag**	**Attached Position**	**ALR-9800 reader**	**Mecury4 reader**
**Sontec Metal Tag**	Side	87%	90%
	Top	83%	83%
**ALR ‘M’ Tag on foam**	Side	86%	88%
	Top	81%	85%
